# Supplementary material for: Content-rich biological network constructed by mining PubMed abstracts
Source: BMC Bioinformatics. 2004 Oct 8;5:147. doi: 10.1186/1471-2105-5-147 (PMC528731; doi:10.1186/1471-2105-5-147)
Supplement: Additional File 5 — The original Chilibot query results of the term "long-term potentiation (LTP)" and 22 other terms, limiting the latest references analyzed to the years 1990, 1995, 2000, and 2004. [file 1471-2105-5-147-S5.bz2 › chilibotAdditionalFile5/ltp2004/html/PI-3K_ACTININ.html]

 


 **PI-3K** and **ACTININ** 
  
Found 9 abstracts in PubMed,  **9 abstracts were retrieved and analyzed**.  


---

 Search Google  |
 PDF files only 
|  EDU domain only 

---

**Interactive relationship** (e.g. stimulation, inhibition, etc)

- These results suggest that PI 3 kinase  [ **PI-3K** ]  binds to alpha  **actinin**  and regulates cytoskeletal reorganization.  Ref: 8093010 Biochem J, 1994
- We have found that alpha  **actinin**  copurifies with PI 3 kinase  [ **PI-3K** ]  from bovine thymus.  Ref: 8093010 Biochem J, 1994
- This coprecipitation was observed even after depolymerization of actin fibres, suggesting that PI 3 kinase  [ **PI-3K** ]  binds directly to alpha  **actinin** .  Ref: 8093010 Biochem J, 1994
- In cell separation, the signaling between the IGF IR kinase and actin required phosphatidylinositol 3 PI 3 kinase  [ **PI-3K** ]  generated phospholipids but not MAP kinases and was mediated by alpha  **actinin** .  Ref: 12356918 J Cell Sci, 2002
- Further evidence demonstrated that activation of PI 3 kinase  [ **PI-3K** ]  by PDGF induced a decrease in the association of alpha  **actinin**  with the integrin beta subunit,.  Ref: 10931873 J Cell Biol, 2000
- Expression of constitutively active Cdc42 induced the formation of FAK PI 3 kinase  [ **PI-3K** ]  and vinculin alpha  **actinin**  complexes whereas active Rac1 induced only a vinculin alpha  **actinin**  complex.  Ref: 11856170 Cell Microbiol, 2002

**Parallel relationship** (e.g. studied together, co-existance, homology, etc.)

- In addition, anti alpha  **actinin**  antibody coprecipitates PI 3 kinase  [ **PI-3K** ]  activity.  Ref: 8093010 Biochem J, 1994
- The antibody against PI 3 kinase  [ **PI-3K** ]  85 kDa subunit p85 also co immunoprecipitates alpha  **actinin**  from lysates of NIH 3T3 cells.  Ref: 8093010 Biochem J, 1994
- Phosphorylation of focal adhesin kinase FAK at Tyr397 and the formation of complexes between FAK and PI 3 kinase  [ **PI-3K** ]  and between alpha  **actinin**  and vinculin were found to correlate with type 1 pilus mediated bacterial invasion.  Ref: 10856226 EMBO J, 2000
- The invasion mechanism requires actin polymerization, focal adhesion kinase phosphorylation and PI 3 kinase  [ **PI-3K** ]  activation as well as the formation of FAK PI 3 kinase  [ **PI-3K** ]  and downstream vinculin alpha  **actinin**  complexes.  Ref: 11856170 Cell Microbiol, 2002
- Aggregation 60 80% of control was restored if 1 unit mL thrombin was added, but there was no corresponding redistribution of actin binding protein, talin, vinculin, alpha  **actinin** , GPIIb IIIa, PECAM, PI3 kinase  [ **PI-3K** ] , and pp60c src to the cytoskeleton.  Ref: 7626626 Biochemistry, 1995
- The addition of 0.2 unit mL thrombin caused aggregation dependent redistribution of cytoskeletal proteins actin binding protein, talin, vinculin, and alpha  **actinin** , glycoproteins GPIIb IIIa, PECAM, and signaling molecules PI3 kinase  [ **PI-3K** ] , pp60c src to the cytoskeletal fraction of platelets.  Ref: 7626626 Biochemistry, 1995
- Functional role of alpha  **actinin** , PI 3 kinase  [ **PI-3K** ]  and MEK1 2 in insulin like growth factor I receptor kinase regulated motility of human breast carcinoma cells.  Ref: 12356918 J Cell Sci, 2002
- We suggest that the signaling pathway from the IGF IR kinase through the PI 3 kinase  [ **PI-3K** ]  to alpha  **actinin**  participates in the rapid organization of actin into microspikes at the cell cell junctions and leads to active cell separation.  Ref: 12356918 J Cell Sci, 2002
